# Evolution of Chernobyl Corium in Water: Formation of Secondary Uranyl Phases

**DOI:** 10.3390/ma16134533

**Published:** 2023-06-22

**Authors:** Vladislav V. Gurzhiy, Boris E. Burakov, Bella Yu. Zubekhina, Anatoly V. Kasatkin

**Affiliations:** 1 Department of Crystallography, Institute of Earth Sciences, St. Petersburg State University, University Emb. 7/9, 199034 St. Petersburg, Russia; 2 Ioffe Institute, 26, Politekhnicheskaya, 194021 St. Petersburg, Russia; burakov@peterlink.ru; 3 Japan Atomic Energy Agency, 2–4 Shirakata, Tokai-mura, Ibaraki 319-1195, Japan; zubekhina.bella@jaea.go.jp; 4 Fersman Mineralogical Museum of the Russian Academy of Sciences, Leninskiy pr. 18, 2, 119071 Moscow, Russia; anatoly.kasatkin@gmail.com

**Keywords:** Chernobyl, corium, uranyl, mineral, becquerelite, phurcalite, topology, crystal structure, X-ray diffraction, Fukushima Daiichi NPP

## Abstract

Two crystalline phases, which are analogues of common secondary uranyl minerals, namely, becquerelite (Ca[(UO_2_)_6_O_4_ (OH)_6_]·8H_2_O) and phurcalite (Ca_2_[(UO_2_)_3_O_2_ (PO_4_)_2_]·7H_2_O) were identified on the surface of a Chernobyl corium-containing sample affected by hydrothermal alteration in distilled water at 150 °C for one year. Phases were characterized using Single-Crystal X-ray Diffraction Analysis (SCXRD) as well as optical and scanning electron microscopy. Features of the structural architecture of novel phases, which come from the specific chemical composition of the initial fragment of Chernobyl sample, are reported and discussed. Precise identification of these phases is important for modelling of severe nuclear accidents and their long-term consequences, including expected corium–water interaction processes at three damaged Units of the Nuclear Power Plant Fukushima Daiichi.

## 1. Introduction

A severe nuclear accident at the 4-th Unit of the Chernobyl Nuclear Power Plant (ChNPP) on 26 April 1986 was characterized with high-temperature interactions between U-oxide nuclear fuel, zircaloy cladding, and construction materials such as steel, serpentine and concrete [1]. Products of corium formation and solidification in the form of solid solutions “UO_2_-ZrO_2_” with different U/Zr ratio were identified in the matrices of so-called Chernobyl “lava” and “hot” particles [2,3]. In addition, corium products were discovered recently in the matrix of an unusual material which consisted of mainly molten and oxidized steel [4]. Such a material was formed during an initial very high-temperature (at least 2400–2600 °C) stage of the accident and it was injected into room 305/2 (right below the reactor core) where it rapidly solidified without interaction with silicate construction material (serpentine and concrete). According to a very cautious estimate, room 305/2 contains about 60 tons of the fuel [5]. 

It was found (for the first time in 1990) that matrices of Chernobyl “lava” interact with the environment. This process is accompanied with the formation of uranyl-phases such as UO_4_·4H_2_O; UO_3_·2H_2_O; UO_2_·CO_3_; Na_4_ (UO_2_) (CO_3_)_3_, etc. [6,7]. Moreover, the formation of uranyl phases, as assumed, could happen on the surface of some “lava” samples stored under laboratory conditions without humidity control [3,8].

The experimental study of the chemical alteration of Chernobyl corium and “lava” is very important in order to model behavior of these highly radioactive materials over long periods of time [9,10,11]. The information obtained can be applied to predict properties of molten fuel materials contacting water since 2011 at Units#1, 2 and 3 of the Fukushima Daiichi Nuclear Power Plant (F-1 NPP).

Herein, we report the results of precise phase identifications of two uranyl compounds, which were formed on the surface of the Chernobyl sample collected in room 305/2 of the Chernobyl “Shelter” [4] and used in previous experiments on hydrochemical alteration [10]. New-formed phases were characterized using several experimental techniques including Single-Crystal X-ray Diffraction Analysis (SCXRD) as well as optical and scanning electron microscopy. Features of the structural architecture of novel phases, which come from the specific chemical composition of the initial fragment of the Chernobyl sample, are reported and discussed.

## 2. Materials and Methods

### 2.1. Chernobyl Corium-Containing Sample

**Figure 1:**
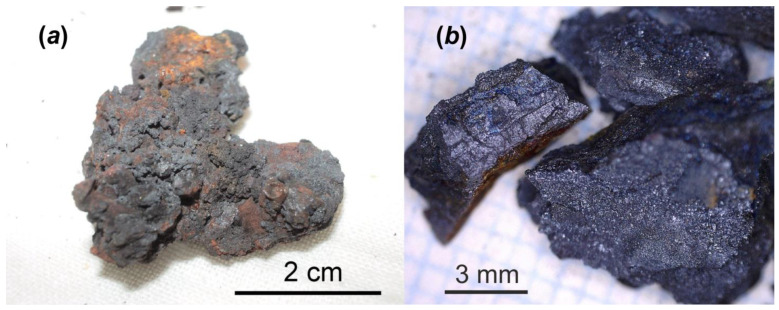
One of the highly radioactive samples consisted of molten and oxidized steel and corium. It was collected by V.A. Zirlin and L.D. Nikolaeva in room 305/2 (right below former reactor core) of the Chernobyl “Shelter” in 1990: general view (**a**); and small broken fragments prepared for alteration test (**b**).

The Chernobyl corium-containing sample (Figure 1) consisted of mainly Fe_3_O_4_ and inclusions of solid solutions “UO_2_-ZrO_2_” (i.e., corium solidification products) with a broad range of U/Zr ratio and was used for chemical alteration experiment in distilled water at 150 °C for one year. Details about chemical and phase composition of this sample have been reported before [4]. The main interest to study this particular type of Chernobyl highly radioactive sample is related to evaluating of physico-chemical durability of corium–steel interaction products over a long time in water under increased temperature. It is assumed that similar materials can be discovered in the near future at Units #1, 2 and 3 of F-1 NPP.

### 2.2. Hydrothermal Alteration Experiment

The 0.15-g fragment of the Chernobyl corium-containing sample and 10 mL of distilled water were placed in a steel autoclave equipped with a 25-mL Teflon liner. The experiment was carried out at a temperature of 150 °C and lasted about a year.

**Figure 2:**
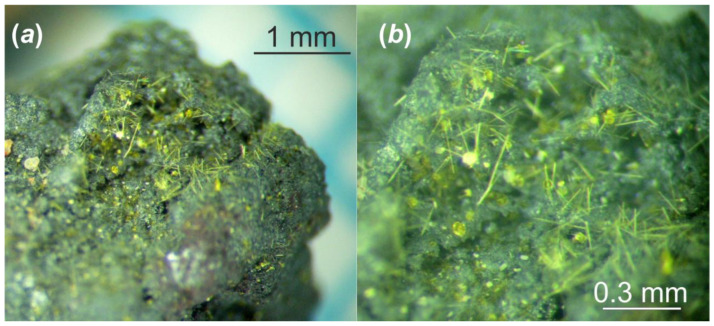
The fragment of the Chernobyl corium-containing sample after hydrothermal alteration at (150 °C in distilled water for 1 year): general view (**a**); and its magnified image (**b**).

**Figure 3:**
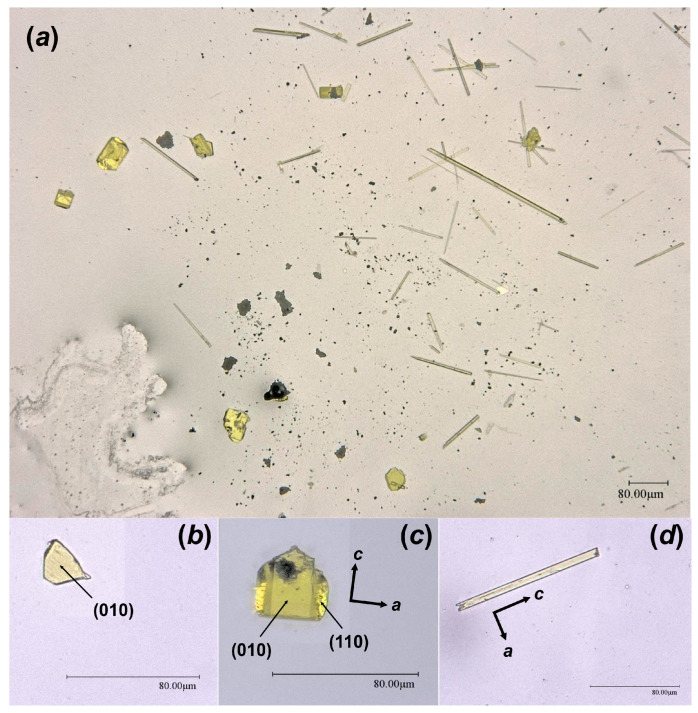
Crystalline phases collected from the surface of the Chernobyl corium-containing sample after the hydrothermal alteration experiment (**a**); examined single crystals of **Bqr_1** (**b**), **Bqr_2** (**c**), and **Phu** (**d**) with shown indexed faces and crystallographic axes orientation.

As the result of this hydrothermal experiment, a highly altered sample of corium-containing material was obtained, the surface of which was covered with yellowish crystals of various sizes and shapes (Figure 2). According to the visual observation of secondary phases using an optical microscope under polarized and cross polarized light, three types of morphologies were found: prismatic, lamellar and flattened needle-like crystals (Figure 3). Pictures of the secondary phases were collected using a digital microscope, Keyence VHX-1000. Further SCXRD studies showed that lamellar (**Bqr_1**) and prismatic (**Bqr_2**) crystals belong to the same structural type, an analog of the uranyl-oxide hydroxy-hydrate mineral becquerelite (Ca[(UO_2_)_6_O_4_ (OH)_6_]·8H_2_O) [12] (Figure 3b,c). Despite morphological differences, both types of crystals are flattened on {010}. Needle-like crystals (**Phu**) appeared to be analogs to another secondary U-bearing mineral, phurcalite (Ca_2_[(UO_2_)_3_O_2_ (PO_4_)_2_]·7H_2_O [13,14]) (Figure 3d).

### 2.3. Chemical Composition

The chemical analyses were carried out with a Hitachi FlexSEM 1000 scanning electron microscope equipped with EDS Xplore Contact 30 detector and Oxford AZtecLive STD system of analysis. Analytical conditions were: accelerating voltage 20 kV and beam current 5 nA. Only Ca, Mn, P, Si, U and O were recorded in **Phu**; Ca, U and O–in **Bqr**. Contents of other elements with atomic numbers higher than that of beryllium were below the detection limits. The following standards and X-ray lines were used: Ca–CaF_2_, K_α_; Mn–Mn_2_SiO_4_, K_α_; Si–SiO_2_, K_α_; P–NdP_5_O_14_, K_α_; U–UO_2_, M_β_.

The chemical composition of **Bqr** is (wt.%, mean of five spots, H_2_O content calculated based on structure): CaO 2.77, UO_3_ 87.32, H_2_O 10.07, total 100.16. The empirical formula based on 30 O *apfu* is Ca_0.97_U^6+^_6.01_H_22_O_30_, or, taking into consideration the structural data, Ca_0.97_U^6+^_6.01_O_16_ (OH)_6_ ·8H_2_O.

The chemical composition of **Phu** is (wt.%, mean of seven spots, H_2_O content calculated based on structure): CaO 8.69, MnO 0.21, SiO_2_ 0.43, P_2_O_5_ 10.89, UO_3_ 69.87, H_2_O 8.71, total 98.80. The empirical formula based on 23 O *apfu* is Ca_1.92_Mn_0.04_P_1.91_Si_0.09_U^6+^_3.03_H_14_O_23_, or, taking in consideration the structural data, (Ca_1.92_Mn_0.04_)_Σ1.96_U^6+^_3.03_ (P_1.91_Si_0.09_)_Σ2.00_O_16_ · 7H_2_O.

### 2.4. Single-Crystal X-ray Diffraction Studies

Single crystals of **Bqr_1**, **Bqr_2** and **Phu** were selected under an optical microscope in polarized light, coated in oil-based cryoprotectant and mounted on a cryoloops. The diffraction data were collected using a Rigaku XtaLAB Synergy S X-ray diffractometer operated with a monochromated microfocus MoKα tube PhotonJet-S (λ = 0.71073 Å) at 50 kV and 1.0 mA and equipped with a CCD HyPix 6000HE hybrid photon-counting detector [15]. The frame width was 1.0^o^ in ω, and exposures ranged from 12 to 110 s for each frame. CrysAlisPro software [16] was used for the integration and correction of diffraction data for polarization, background and Lorentz effects, as well as for absorption correction. An empirical absorption correction based on spherical harmonics implemented in the SCALE3 ABSPACK algorithm was applied. The unit-cell parameters (Table 1) were refined using the least-squares techniques. The structures were solved by a dual-space algorithm and refined using SHELX programs [17,18], incorporated in the OLEX2 program package [19]. The final model included coordinates and anisotropic displacement parameters for all non-H atoms. H atoms were localized from different Fourier maps and were included in the refinement with bond lengths and isotropic displacement parameters restraints. The crystal structures of **Bqr_1** and **Bqr_2** were refined as two-component inversion twins with statistically equal contribution of components (0.54 (3)/0.46 (3) and 0.56 (3)/0.44 (3), respectively). Supplementary crystallographic data were deposited in the Inorganic Crystal Structure Database (ICSD) and can be obtained by quoting the CSD 2256603-2256605 via www.ccdc.cam.ac.uk/structures/ (see Supplementary Materials).

## 3. Results

The mineral becquerelite was discovered a century ago [20], and its chemical composition and lattice parameters were then additionally reported [21,22]. The crystal structure of becquerelite was first reported by Piret-Meunier and Piret [12]. Later, the structural model of becquerelite was refined to better values of convergence factors [23,24] and spectroscopic studies have been performed [25,26,27]. Our SCXRD investigations confirm known structural models, and atom arrangements; naming from the latest model reported by Burns and Li [24] was taken as a starting set in the current work. It should be noted that all previous studies described a becquerelite unit cell in a non-conventional *Pn*2_1_*a* setting (Table 2). Structural models of **Bqr_1** and **Bqr_2** are reported in a standard setting, which corresponds to the *mm*2 point group.

The crystal structure of **Bqr** contains of six crystallographically independent U^6+^ cations. Each U^6+^ cation is strongly bonded to two O^2-^ atoms, forming almost linearly within 7° O^2-^≡U^6+^≡O^2-^ uranyl cations (*Ur*) with U–O_Ur_ bond lengths ranging from 1.724 (16) to 1.854 (19) Å (Table 3 and Table 4). All six *Ur* ions are equatorially coordinated by five O atoms, which results in the formation of pentagonal bipyramids (U–O_eq_ = 2.16 (2)–2.78 (3) Å). Besides, three out of five equatorial bonds are accounted for by O atoms of the hydroxyl groups. There is also one crystallographically unique Ca^2+^ cation in the structure of **Bqr**, which is coordinated by four O_Ur_ atoms and another four O atoms of H_2_O molecules with Ca–O = 2.36 (2)–3.049 (18) Å to form square antiprism coordination polyhedron.

**Figure 4:**
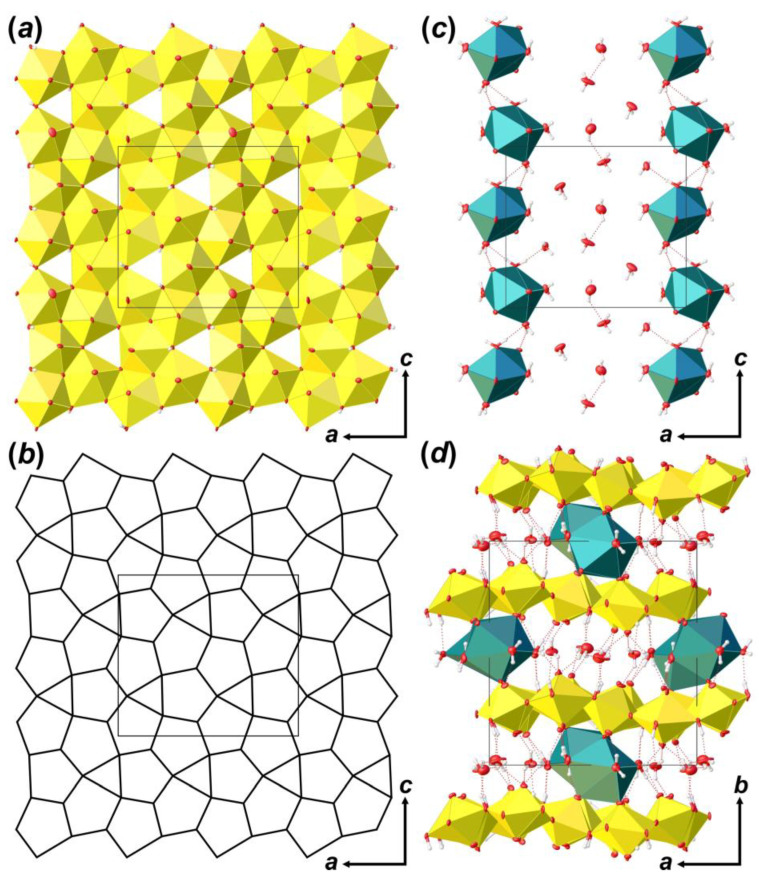
The crystal structure of **Bqr**: polyhedral representation of the uranyl-hydroxy-oxide layer (**a**); its anion-topology (**b**); fragment of the interlayer space (**c**); and the structure of **Bqr** projected along the [001] (**d**). Legend: U polyhedra = yellow; Ca polyhedra = cyan; O atoms are red; hydrogen atoms are small white circles; H-bonds = dashed red lines; thermal ellipsoids are shown at the 50% probability level.

Coordination polyhedra of U atoms share equatorial edges and vertices to form layers of [(UO_2_)_6_O_4_ (OH)_6_]^2–^ composition that are arranged parallel to (010) (Figure 4a). The layer of uranyl pentagonal bipyramids can be described in terms of anion-topology as formed by triangles and pentagons [34] with a …PDPD… stacking sequence of polygonal chains [35,36,37] and 5^4^3^1^ cyclic symbol [38,39] (Figure 4b). All pentagons are occupied by *Ur*, while all triangles are empty. This type of polygon arrangement is attributed to the so-called protasite or α-U_3_O_8_ anion-topology, which was also found in the structures of a number of minerals and synthetic compounds like protasite [23], billietite [23], compreignacite [40], masuyite [41], agrinierite [42], α-U_3_O_8_ [43], Na_2_[(UO_2_)_3_O_3_ (OH)_2_] [44], etc. In between the U-bearing layers, one crystallographically non-equivalent Ca^2+^ cation and eight H_2_O molecules are arranged (Figure 4c). Ca-centered polyhedra are organized in 1D units that are stretched along the [001]. Four out of eight H_2_O molecules are arranged in the coordination sphere of Ca^2+^ cations, and four molecules fill the gap between the chains of Ca-polyhedra and link with U-layers and Ca-chains only through the system of H-bonds (Figure 4d; Table 5). It should be noted that the system of H-bonds in the structure of **Bqr**, which was revealed after the assignment of H atoms sites, in general, corresponds to that proposed by Burns and Li [24]. However, several discrepancies can be found; for instance, OW24⋯O8 instead of OW24⋯OW27, or OW30⋯O10 instead of OW30⋯OW24 in **Bqr** and [24], respectively.

The mineral phurcalite was discovered by Deliens and Piret [13], who have reported on its orthorhombic symmetry, chemical composition and its lattice parameters. The structural model of phurcalite was reported the same year [14]. Later, the structure of phurcalite was refined several times for different specimens from various localities (Table 2) [28,29,30]. The most recent study reports on the H-bonding system, which was determined by a combination of SCXRD and modern computational methods [31]. The structural model of phurcalite reported in [31] was taken as a starting set of atoms in the current work.

**Figure 5:**
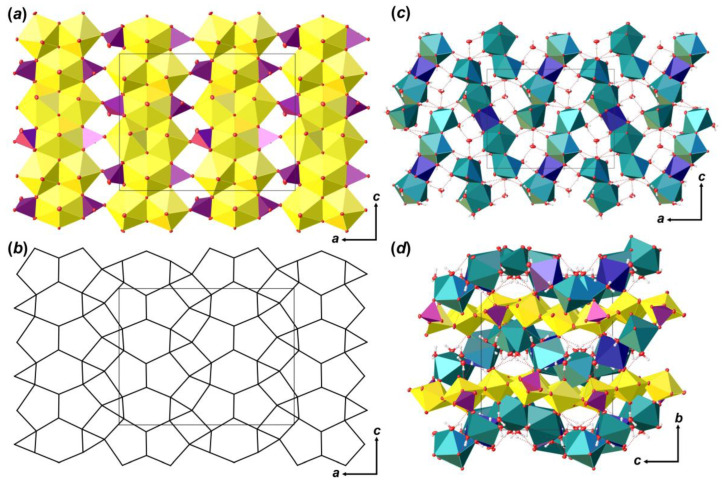
The crystal structure of **Phu**: polyhedral representation of the uranyl phosphate layer (**a**); its anion-topology (**b**); fragment of the interlayer space (**c**); and the structure of **Phu** projected along the [100] (**d**). Legend: see Figure 4; Mn octahedra = dark blue; P tetrahedra = violet.

**Figure 6:**
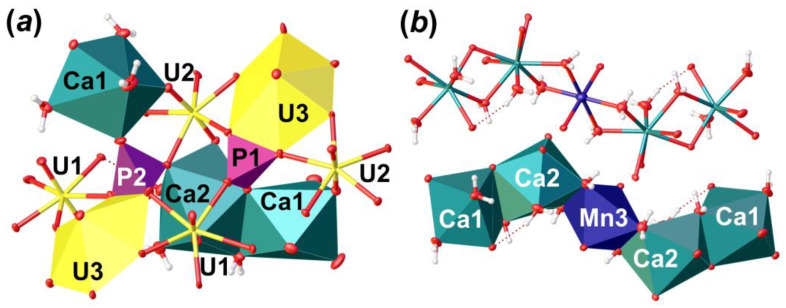
The crystal structure of **Phu**: an arrangement of phosphate tetrahedra (**a**); and an organization of Ca-Mn pentamers from the interlayer space in polyhedral and ellipsoidal representation (**b**). Legend: see Figure 5.

The crystal structure of **Phu** (Figure 5) contains three crystallographically independent U^6+^ cations. The U–O_Ur_ bond lengths range from 1.798 (3) to 1.822 (3) Å (Table 6). *Ur*1 and *Ur*2 ions are equatorially coordinated by five O atoms, which results in the formation of pentagonal bipyramids (U–O_eq_ = 2.252 (3)–2.512 (3) Å). The *Ur*3 ion is equatorially coordinated by six O atoms to form hexagonal bipyramid (U–O_eq_ = 2.221 (3)–2.790 (3) Å). There are two crystallographically non-equivalent P^5+^ cations in the structure of **Phu**, tetrahedrally coordinated by four O atoms each with <P–O> = 1.535 and 1.546 Å for P1 and P2, respectively. It is of interest that P-centered tetrahedra has slightly different coordination environment (Figure 6a). [P1O4]^3–^ oxyanion shares an equatorial O2···O6 edge with *Ur*3 hexagonal bipyramid, an equatorial O11 vertex with *Ur*3 cation, and a bridged O13 atom, which is a part of a common O13···H_2_O20 edge between Ca1 and Ca2 polyhedra. The [P2O4]^3–^ oxyanion also shares an equatorial O8···O15 edge with *Ur*3 hexagonal bipyramid, O18 atom with Ca1 coordination polyhedron, and O9 atom, which is a part of O5···O9 edge common between Ca2 and U2 coordination polyhedra. Slight deficiency of bond valence sums (BVS) for the P2 site, along with a slight elongation of the <P2–O> bond length (compared to that for P1; Table 6), and the results of chemical analysis, all indicate the presence of less than 0.1 Si atoms per formula unit (p.f.u.) in the structure of **Phu**; this allows considering P2 site as (P_0.91_Si_0.09_). Such a distribution most likely comes from the fact that the P1 site is more tightly bonded than the P2 site, which prevents a larger Si cation from occupying it. Similar crystal chemical restrictions for the larger Se^6+^ cations incorporation in tighter S^6+^ sites were observed in a course of phase formation studies in the mixed actinyl sulfate–selenate aqueous systems [45,46,47,48,49,50].

The crystal structure of **Phu** is based on the uranyl phosphate layers of [(UO_2_)_3_O_2_ (PO_4_) (P_0.91_Si_0.09_O_4_)] compositions (Figure 5a), which are arranged parallel to (010). Anion-topology of the layer corresponds to the phosphuranylite type with 6^1^5^2^4^2^3^2^ cyclic symbol [38,39], and can be described as formed by triangles, squares, pentagons and hexagons [34], where all hexagons and pentagons are occupied by *Ur*, all triangles are occupied by phosphate oxyanions (Figure 5b), and all squares stay vacant. This is one of the most common topological types of U-bearing 2D units. About 50 compounds of both natural and synthetic origin and various chemical compositions are known nowadays (e.g. [34,51,52,53,54,55,56,57]). Layers are formed by the large number of chains of dimers of edge-shared uranyl pentagonal bipyramids that are further connected by edge-shared U-centered hexagonal bipyramids. Neighbor chains are shifted by the half period as they lengthen, so that hexagonal bipyramids are arranged in front of dimers of pentagonal bipyramids. In these places, the chains are linked into a layer through the phosphate tetrahedra, which share an edge with hexagonal bipyramid from one chain, and a vertex with pentagonal bipyramid from a neighbor chain.

There are two non-equivalent Ca^2+^ sites, one Mn^2+^ site and six H_2_O molecules arranged in between the uranyl phosphate layers (Figure 5c). Ca1 site is surrounded by three H_2_O molecules and two O_Ur_ atoms, and two O atoms are shared with two distinct phosphate groups with <Ca1–O> = 2.424 Å. Ca2 site is surrounded by four H_2_O molecules, two O_Ur_ atoms, and two O atoms are shared with two distinct phosphate groups with <Ca1–O> = 2.498 Å. Ca1 and Ca2 coordination polyhedra share common O13···H_2_O20 edge to form dimer. The Mn3 site occupies an inversion center, which is arranged between two neighbor dimers of Ca-centered polyhedra. This site represents a rather classical octahedron surrounded by four H_2_O molecules (Mn3–H_2_O = 2.207 (3)–2.266 (4) Å) in the equatorial plane and another two apical O_Ur_ atoms with slightly elongated bonds (Mn3–O_Ur_12 = 2.387 (3) Å), which can fit any of divalent cations. In the case of **Phu** crystal, an electron density peak of *c.a*. 1.1 *e*/Å^3^ was arranged in this site. Chemical analyses showed the presence of Mn in the examined samples, the amount of which corresponds to the site occupancy revealed in a course of SCXRD studies. Moreover, the presence of a cation at the Mn3 site results in a formation of the Ca1-Ca2-Mn3-Ca2-Ca1 pentamers via sharing common edges between Ca- and Mn-centered coordination polyhedra (Figure 6b). Pentamers are stretched along c.a. [102] and [-102] and separated by an additional H_2_O23 molecule, which links uranyl phosphate layers and pentamers only through H-bonds.

## 4. Discussion

Analogues of becquerelite discussed within this paper do not significantly differ in chemical composition and crystal structure from the previously studied natural samples. However, we report the crystal structure of becquerelite in the standard *Pna*2_1_ setting for the first time, along with all H atom site assignments, which allow us to demonstrate the branchy H-bonding system. Investigation of phurcalite analogs have demonstrated differences in the structural architecture of known natural and obtained synthetic phases. Thus, the new octahedral site between the uranyl phosphate layers occupied by Mn atoms was found. It can be assumed that incorporation of a cation into the Mn3 site and the formation of pentamers result in a stronger linkage of uranyl phosphate layers into 3D structure. Compensation of an additional positive charge that comes with the incorporation of Mn^2+^ cations occurs due to the heterovalent isomorphism of Si^4+^ cations in the P^5+^ sites. Additional compensation, if needed, may come from the replacement of H_2_O16 and H_2_O19 molecules, which form an equatorial plane of Mn-centered octahedron and are included in the coordination sphere of Ca_2_ cations, by O^2–^ anions or OH^–^ groups. Thus, the formula of the studied **Phu** crystal according to the SCXRD and SEM data could be given as Ca_2_Mn_0.03_[(UO_2_)_3_O_2_ (PO_4_) (P_0.94_Si_0.06_O_4_)]·7H_2_O. It is of interest that, in previous studies of natural phurcalite crystals, no additional cation sites except for Ca1 and Ca2 have been found within the interlayer space. This example shows that a possible re-investigation of phurcalite mineral samples is needed to check if any additional cations that may occupy the Mn3 octahedral site.

The Chernobyl corium-containing sample used in this research is a product of high temperature co-melting of U-oxide fuel, zircaloy cladding, steel, serpentine and concrete [4]. As a result, it has a unique and complex chemical and phase compositions. It can explain the composition of uranyl phases formed during the alteration experiment. Uranium comes from the relicts of overheated nuclear fuel (UO_x_) and corium inclusions (U-Zr-O with high U/Zr ratio), which is easy to oxidize to the 6+ state in aqueous solutions. Calcium and Si come from the concrete. Phosphorus and Mn, most likely, come from construction steel of 10HSND grade (10XCHД in Russian), used in the low basic reactor plate “OR” (“OP” in Russian). This steel contains 0.5–0.8 wt.% Mn and up to 0.035 wt.% P [58].

**Figure 7:**
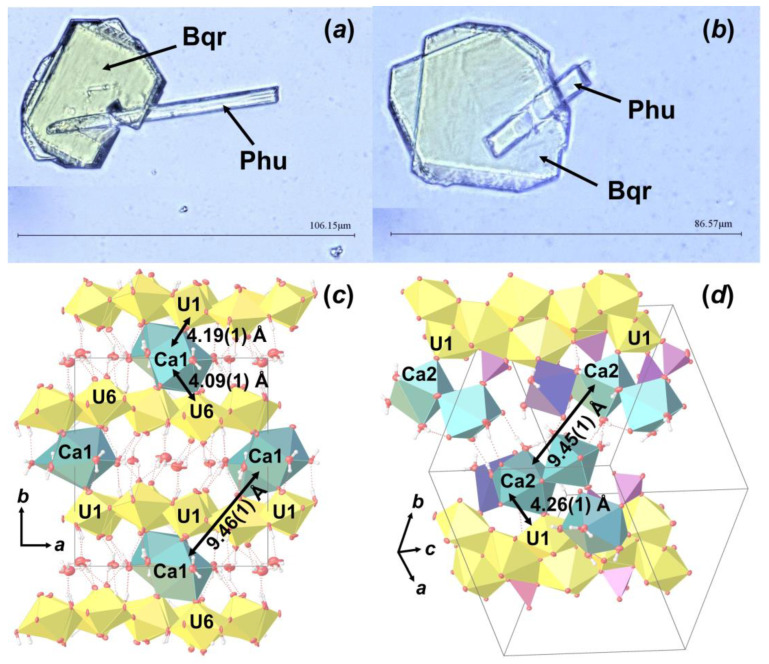
Intergrowth of lamina **Bqr** crystals with needle-like **Phu** (**a**,**b**); and the arrangement of **Bqr** (**c**) and **Phu** (**d**) crystal structures, in which projections intergrowing occurs. Legend: see Figure 5.

During optical microscopy studies of the alteration products, several intergrowth of lamina and needle crystals were found (Figure 7a,b). SCXRD experiments showed that these are intergrowths of **Bqr** and **Phu**, which can be described as follows: rotation of **Phu** unit cell relative to the **Bqr** by 142.83 ° around the c.a. [−0.25 0 1] axis, which corresponds to the approximate coincidence of the [001] direction in the structure of **Bqr** with the [−1−11] direction in the structure of **Phu** (Figure 7b,c). In these directions both structures have similar arrangement of Ca polyhedra and U bipyramids neighbor to them. Hence, one can assume that intergrowing relates exactly to these structural fragments. To our knowledge, this is the first reported evidence of becquerelite and phurcalite intergrowth.

## 5. Conclusions

Two analogues of common secondary uranyl minerals, becquerelite and phurcalite, formed on the surface of a Chernobyl corium-containing sample affected by hydrothermal alteration were identified and studied in detail. The results obtained are proposed to be included into a database for modelling of long-term behavior of corium–steel interaction products forming as a consequence of severe nuclear accidents.

The fact that, during hydrothermal experiment, only crystals with dense polymerization of uranyl polyhedra (i.e., that share common edges) were obtained, confirms our recently made assumption [56,57,59,60] that such structures should not crystalize at ambient temperature and an additional energy source is needed to obtain phases with dense architecture, while uranyl minerals and compounds with sparse structural units (i.e., that share only common vertices) can crystallize from aqueous solutions at ambient conditions.

The results of reported studies are important not only for predicting corium aging in anticipation of decommissioning, but also for evaluating the stability of corium, spent fuel, and cemented U-bearing wastes under temporary storage and final repository conditions [61,62,63].

The chemical stability of the corium should be modelled taking into account potential formation of secondary uranyl phases and their further chemical and physical alteration. Short-term leach tests do not provide enough time for the growth of secondary mineral-like phases. Therefore, such an important process is usually not taken into account in the models [64,65,66,67,68,69], although uranyl phases are obviously less stable than U oxide.

It is known from the model experiments that analogues of becquerelite are formed during the aging of spent fuel [70]. Thus, one can assume that the initial chemical forms of uranium are less important in most cases for the formation of these phases than particular oxidizing conditions and properties of the environment [71,72,73,74,75,76,77]. Corium, which possibly formed at F-1 NPP may differ chemically from Chernobyl corium [4,10,78], but the products of its alteration in water would be similar.

## Figures and Tables

**Table 1:** Crystallographic data for lamellar (**Bqr_1**) and prismatic (**Bqr_2**) crystal analogs of becquerelite, and for needle-like (**Phu**) crystal analog of phurcalite.

Sample	Bqr_1	Bqr_2	Phu
Crystal System	Orthorhombic	Orthorhombic	Orthorhombic
Space group	*Pna*2_1_	*Pna*2_1_	*Pbca*
*a* (Å)	13.8517 (5)	13.9073 (6)	17.4042 (3)
*b* (Å)	14.9553 (6)	15.0023 (6)	16.0025 (3)
*c* (Å)	12.3753 (5)	12.4269 (6)	13.5595 (2)
*V* (Å^3^)	2563.62 (17)	2592.8 (2)	3776.47 (11)
Molecular weight	1970.43	1970.43	1239.67
μ (mm^–1^)	38.083	37.655	26.508
Temperature (K)	293 (2)	293 (2)	293 (2)
*Z*	4	4	8
*D*_calc_ (g/cm^3^)	5.105	5.048	4.361
Crystal size (mm^3^)	0.030 × 0.020 × 0.002	0.052 × 0.034 × 0.021	0.120 × 0.010 × 0.001
Radiation	Mo*K*α	Mo*K*α	Mo*K*α
Total reflections	23016	14077	40125
Unique reflections	5722	5031	5503
Angle range 2*θ* (°)	6.48–55.00	6.46–55.00	6.46–60.00
Reflections with |*F*_o_| ≥ 4σ*_F_*	4698	4284	4834
*R*_int_	0.0546	0.0394	0.0428
*R*_σ_	0.0536	0.0481	0.0278
*R*_1_ (|*F*_o_| ≥ 4σ*_F_*)	0.0392	0.0352	0.0203
*wR*_2_ (|*F*_o_| ≥ 4σ*_F_*)	0.0762	0.0762	0.0360
*R*_1_ (all data)	0.0550	0.0452	0.0273
*wR*_2_ (all data)	0.0803	0.0801	0.0373
*S*	1.058	1.053	1.034
*ρ*_min_, *ρ*_max_, *e*/Å^3^	−3.355, 2.194	−2.203, 1.142	−0.919, 1.017
CSD	2256603	2256604	2256605

*R*_1_ = Σ||*F*_o_| − |*F*_c_||/Σ|*F*_o_|; *wR*_2_ = {Σ[*w* (*F*_o_^2^ − *F*_c_^2^)^2^]/Σ[*w* (*F*_o_^2^)^2^]}^1/2^; *w* =1/[σ^2^ (*F*_o_^2^) + (a*P*)^2^ + b*P*], where *P* = (*F*_o_^2^ + 2*F*_c_^2^)/3; *s* = {Σ[*w* (*F*_o_^2^ − *F*_c_^2^)]/ (*n* − *p*)}^1/2^ where *n* is the number of reflections and *p* is the number of refinement parameters.

**Table 2:** Comparison of the becquerelite and phurcalite unit cell parameters reported previously and in the current work.

Becquerelite						
Reference	[21]	[12]	[23]	[24]	Bqr_1	Bqr_2
Sp. Gr.		*Pn*2_1_*a*	*Pn*2_1_*a*	*Pn*2_1_*a*	*Pna*2_1_	*Pna*2_1_
a, Å	13.920 (5)	13.86 (2)	13.8378 (8)	13.8527 (5)	13.8517 (5)	13.9073 (6)
*b*, Å	12.450 (5)	12.30 (1)	12.3781 (12)	12.3929 (4)	14.9553 (6)	15.0023 (6)
*c*, Å	15.090 (5)	14.92 (3)	14.9238 (9)	14.9297 (5)	12.3753 (5)	12.4269 (6)
*V*, Å^3^	2620.79	2543.53	2556.23	2563.2 (1)	2563.62 (17)	2592.8 (2)
Phurcalite *						
Reference	[13,14]	[28]	[29]	[30]	[31]	Phu
a, Å	17.426 (3)	17.44 (2)	17.415 (2)	17.3785 (9)	17.4652 (5)	17.4042 (3)
*b*, Å	16.062 (3)	15.87 (2)	16.035 (3)	15.9864 (8)	16.0068 (5)	16.0025 (3)
*c*, Å	13.592 (3)	13.56 (3)	13.598 (3)	13.5477 (10)	13.5710 (4)	13.5595 (2)
*V*, Å^3^	3804	3753	3797 (2)	3763.8 (4)	3793.9 (2)	3776.47 (11)

* All structural models have been refined in the *Pbca* space group.

**Table 3:** Selected geometrical parameters in the structures of **Bqr_1** and **Bqr_2**: bond lengths, Å; and bond-valence sums (BVS *, values are given in valence units).

	Bqr_1		Bqr_2	
Bond		BVS		BVS
U1–O1	1.80 (2)	1.622	1.796 (18)	1.634
U1–O2	1.829 (19)	1.534	1.823 (18)	1.552
<U1–O_Ur_>	1.815		1.810	
U1–O13	2.200 (18)	0.750	2.248 (16)	0.684
U1–O14	2.16 (2)	0.811	2.220 (17)	0.722
U1–OH17	2.64 (2)	0.321	2.64 (2)	0.321
U1–OH18	2.42 (3)	0.491	2.42 (3)	0.491
U1–OH19	2.45 (2)	0.464	2.42 (2)	0.491
<U1–O_eq_>	2.374	Σ (U1) = 5.993	2.390	Σ (U1) = 5.896
U2–O3	1.790 (14)	1.653	1.797 (13)	1.631
U2–O4	1.813 (12)	1.582	1.803 (11)	1.613
<U2–O_Ur_>	1.802		1.800	
U2–O13	2.254 (18)	0.676	2.249 (16)	0.683
U2–O15	2.252 (19)	0.679	2.253 (17)	0.678
U2–OH17	2.47 (2)	0.446	2.47 (2)	0.446
U2–OH20	2.626 (12)	0.330	2.663 (11)	0.308
U2–OH21	2.37 (2)	0.541	2.422 (19)	0.489
<U2–O_eq_>	2.394	Σ (U2) = 5.908	2.411	Σ (U2) = 5.847
U3–O5	1.85 (2)	1.473	1.854 (19)	1.462
U3–O6	1.77 (2)	1.718	1.780 (18)	1.686
<U3–O_Ur_>	1.810		1.817	
U3–O15	2.265 (18)	0.662	2.223 (17)	0.718
U3–O16	2.27 (2)	0.656	2.250 (17)	0.682
U3–OH18	2.50 (3)	0.421	2.50 (3)	0.421
U3–OH21	2.60 (2)	0.347	2.648 (19)	0.317
U3–OH22	2.33 (2)	0.584	2.37 (2)	0.541
<U3–O_eq_>	2.393	Σ (U3) = 5.862	2.398	Σ (U3) = 5.825
U4–O7	1.826 (13)	1.543	1.821 (11)	1.558
U4–O8	1.816 (13)	1.573	1.808 (11)	1.597
<U4–O_Ur_>	1.821		1.815	
U4–O14	2.24 (2)	0.695	2.28 (2)	0.643
U4–O16	2.25 (3)	0.682	2.19 (2)	0.765
U4–OH18	2.578 (12)	0.362	2.607 (12)	0.343
U4–OH19	2.38 (2)	0.531	2.40 (2)	0.510
U4–OH22	2.42 (2)	0.491	2.43 (2)	0.482
<U4–O_eq_>	2.374	Σ (U4) = 5.876	2.381	Σ (U4) = 5.898
U5–O9	1.79 (2)	1.653	1.811 (17)	1.588
U5–O10	1.741 (19)	1.817	1.724 (16)	1.878
<U5–O_Ur_>	1.766		1.768	
U5–O13	2.270 (17)	0.656	2.248 (15)	0.684
U5–O14	2.29 (2)	0.631	2.218 (19)	0.725
U5–OH17	2.37 (2)	0.541	2.403 (19)	0.508
U5–OH19	2.69 (2)	0.292	2.73 (2)	0.270
U5–OH20	2.40 (3)	0.510	2.44 (3)	0.473
<U5–O_eq_>	2.404	Σ (U5) = 6.101	2.408	Σ (U5) = 6.125

* BVS were calculated using the following parameters: U [32], P, Ca, Mn [33].

**Table 4:** Selected geometrical parameters in the structures of **Bqr_1** and **Bqr_2**: bond lengths, Å; angles, °; and BVS * (v. u.).

	Bqr_1		Bqr_2	
Bond		BVS		BVS
U6–O11	1.795 (19)	1.638	1.787 (16)	1.663
U6–O12	1.842 (18)	1.496	1.852 (15)	1.467
<U6–O_Ur_>	1.819		1.820	
U6–O15	2.180 (18)	0.780	2.245 (16)	0.688
U6–O16	2.18 (2)	0.780	2.29 (2)	0.631
U6–OH20	2.41 (3)	0.501	2.37 (3)	0.541
U6–OH21	2.43 (2)	0.482	2.399 (19)	0.511
U6–OH22	2.78 (3)	0.245	2.76 (2)	0.255
<U6–O_eq_>	2.396	Σ (U6) = 5.921	2.413	Σ (U6) = 5.757
Ca1–O1	2.45 (2)	0.265	2.466 (19)	0.255
Ca1–O3	3.024 (17)	0.065	3.049 (18)	0.061
Ca1–O5	2.43 (2)	0.278	2.43 (2)	0.278
Ca1–O12	2.36 (2)	0.330	2.362 (17)	0.329
Ca1–H_2_O23	2.47 (2)	0.252	2.49 (2)	0.240
Ca1–H_2_O24	2.44 (2)	0.272	2.42 (2)	0.285
Ca1–H_2_O25	2.38 (3)	0.315	2.36 (3)	0.330
Ca1–H_2_O26	2.56 (2)	0.203	2.59 (2)	0.188
<Ca1–O>	2.514	Σ (Ca1) = 1.980	2.521	Σ (Ca1) = 1.968
Angle				
U1–O13–U2	121.9 (8)		120.7 (7)	
U1–OH17–U2	99.2 (7)		99.8 (7)	
U1–OH18–U3	146.2 (6)		147.3 (5)	
U1–O14–U4	123.0 (11)		119.3 (8)	
U1–OH18–U4	101.4 (9)		100.9 (7)	
U1–OH19–U4	143.8 (10)		146.3 (9)	
U1–O13–U5	118.8 (8)		118.3 (7)	
U1–O14–U5	117.4 (11)		119.0 (9)	
U1–OH17–U5	98.6 (8)		98.6 (7)	
U1–OH19–U5	96.9 (8)		96.8 (7)	
U2–O15–U3	116.8 (8)		119.3 (7)	
U2–OH21–U3	101.3 (8)		99.2 (6)	
U2–O13–U5	116.4 (7)		118.1 (7)	
U2–OH17–U5	146.0 (9)		145.4 (8)	
U2–OH20–U5	99.8 (9)		98.0 (7)	
U2–O15–U6	120.0 (8)		117.9 (7)	
U2–OH20–U6	99.2 (9)		99.8 (8)	
U2–OH21–U6	140.1 (10)		140.4 (8)	
U3–O16–U4	117.9 (10)		122.3 (9)	
U3–OH18–U4	99.3 (8)		99.0 (7)	
U3–OH22–U4	145.1 (11)		142.9 (9)	
U3–O15–U6	122.6 (9)		122.1 (8)	
U3–O16–U6	117.3 (11)		114.1 (8)	
U3–OH21–U6	98.0 (8)		97.9 (7)	
U3–OH22–U6	99.3 (9)		99.0 (7)	
U4–O14–U5	119.4 (9)		121.6 (8)	
U4–OH19–U5	100.7 (7)		99.6 (7)	
U4–O16–U6	124.4 (9)		122.8 (8)	
U4–OH22–U6	97.6 (7)		98.1 (7)	
U5–OH20–U6	145.9 (5)		147.3 (5)	

* BVS were calculated using the following parameters: U [32], P, Ca, Mn [33].

**Table 5:** H-bonding system in the structure of **Bqr_1**. The most likely contacts are marked bold.

*D*–H···*A*	*D*–H, Å	H···*A*, Å	*D*···*A*, Å	<*D*H*A*, °
OH groups				
**OH17–HH17···OW29**	0.90	1.87	2.74 (3)	163
OH18–HH18···O5	0.90	2.61	3.05 (4)	110
OH18–HH18···O8	0.90	2.43	2.98 (2)	120
**OH18–HH18···OW25**	0.90	2.26	2.95 (2)	133
**OH19–HH19···OW26**	0.90	2.10	2.95 (3)	157
**OH20–HH20···OW27**	0.85	2.03	2.84 (2)	157
**OH21–HH21···OW28**	0.90	1.75	2.58 (4)	154
**OH22–HH22···OW30**	0.90	2.00	2.82 (3)	152
H_2_O molecules				
**OW23–HW2A···O8**	1.01	2.03	2.95 (3)	151
OW23–HW2A···OW27	1.01	2.50	3.19 (3)	126
**OW23–HW2B···O3**	0.99	2.13	3.09 (2)	162
**OW24–HW2C···O8**	1.04	1.87	2.90 (4)	167
**OW24–HW2D···O10**	1.00	2.10	3.06 (3)	158
**OW25–HW2E···OW30**	0.88	1.91	2.75 (4)	160
**OW25–HW2F···OW23**	0.89	1.96	2.84 (3)	177
**OW26–HW2G···O9**	0.95	2.11	2.97 (4)	150
**OW26–HW2H···O4**	0.95	2.11	3.01 (3)	159
**OW27–HW2I···O2**	0.95	2.18	3.05 (2)	151
OW27–HW2I···O8	0.95	2.53	3.05 (2)	114
**OW27–HW2J···O11**	1.02	1.99	2.94 (2)	154
**OW28–HW2K···O7**	0.85	2.01	2.78 (2)	151
OW28–HW2L···O9	0.94	2.41	3.21 (4)	143
**OW28–HW2L···OW29**	0.94	2.11	2.81 (2)	130
OW29–HW2M···O4	0.79	2.57	3.09 (4)	124
**OW29–HW2M···O7**	0.79	2.13	2.72 (3)	132
**OW29–HW2N···O11**	0.91	2.13	3.00 (3)	162
**OW30–HW3A···O10**	0.92	2.12	3.01 (2)	163
**OW30–HW3B···O4**	0.85	2.17	2.96 (2)	155

**Table 6:** Selected geometrical parameters in the structure of **Phu**: bond lengths, Å; angles, °; and BVS* (v. u.).

Bond		BVS	Bond		BVS
U1–O12	1.798 (3)	1.628	Ca1–18	2.258 (3)	0.424
U1–O14	1.807 (3)	1.600	Ca1–H_2_O22	2.347 (4)	0.341
<U1–O_Ur_>	1.803		Ca1–H_2_O17	2.369 (3)	0.323
U1–O1	2.282 (3)	0.641	Ca1–O13	2.388 (3)	0.308
U1–O3	2.284 (3)	0.638	Ca1–H_2_O20	2.496 (3)	0.237
U1–O11	2.350 (3)	0.562	Ca1–O4	2.502 (3)	0.233
U1–O8	2.425 (3)	0.486	Ca1–O7	2.606 (3)	0.181
U1–O15	2.512 (3)	0.411	<Ca1–O>	2.424	Σ (Ca1) = 2.048
<U1–O_eq_>	2.371	Σ (U1) = 5.967			
			Ca2–O13	2.368 (3)	0.324
U2–O5	1.813 (3)	1.582	Ca2–H_2_O21	2.387 (4)	0.309
U2–O7	1.822 (3)	1.555	Ca2–H_2_O16	2.403 (3)	0.297
<U2–O_Ur_>	1.818		Ca2–O9	2.407 (3)	0.294
U2–O1	2.252 (3)	0.679	Ca2–H_2_O19	2.426 (4)	0.281
U2–O3	2.295 (3)	0.625	Ca2–H_2_O20	2.563 (3)	0.201
U2–O6	2.334 (3)	0.580	Ca2–O5	2.662 (3)	0.158
U2–O2	2.348 (3)	0.564	Ca2–O14	2.764 (3)	0.123
U2–O9	2.451 (3)	0.463	<Ca2–O>	2.498	Σ (Ca2) = 1.988
<U2–O_eq_>	2.336	Σ (U2) = 6.047			
			Mn3–H_2_O16 x2	2.207 (3)	0.326
U3–O10	1.806 (3)	1.603	Mn3–H_2_O19 x2	2.266 (4)	0.283
U3–O4	1.813 (3)	1.582	Mn3–O12 x2	2.387 (3)	0.212
<U3–O_Ur_>	1.810		<Mn3–O>	2.287	Σ (Mn3) = 1.643
U3–O3	2.221 (3)	0.721			
U3–O1	2.238 (3)	0.697	Angle		
U3–O15	2.470 (3)	0.446	U1–O1–U2	110.61 (11)	
U3–O6	2.569 (3)	0.369	U1–O3–U2	109.00 (11)	
U3–O2	2.683 (3)	0.296	U1–O1–U3	122.17 (11)	
U3–O8	2.790 (3)	0.241	U1–O3–U3	122.54 (11)	
<U3–O_eq_>	2.495	Σ (U3) = 5.955	U1–O8–U3	98.23 (10)	
			U1–O15–U3	105.18 (10)	
P1–O13	1.518 (3)	1.304	U2–O1–U3	120.68 (11)	
P1–O11	1.525 (3)	1.282	U2–O2–U3	101.99 (9)	
P1–O6	1.546 (3)	1.216	U2–O3–U3	120.23 (11)	
P1–O2	1.552 (3)	1.198	U2–O6–U3	105.38 (10)	
<P1–O>	1.535	Σ (P1) = 5.000	U1–O11–P1	137.45 (17)	
			U1–O8–P2	141.20 (18)	
P2–O18	1.504 (3)	1.351	U1–O15–P2	141.54 (17)	
P2–O8	1.556 (3)	1.186	U2–O2–P1	135.21 (16)	
P2–O9	1.556 (3)	1.186	U2–O6–P1	147.36 (17)	
P2–O15	1.567 (3)	1.154	U2–O9–P2	131.28 (15)	
<P2–O>	1.546	Σ (P2) = 4.876	U3–O2–P1	97.93 (13)	
			U3–O6–P1	102.86 (13)	
			U3–O8–P2	93.81 (14)	
			U3–O15–P2	106.76 (15)	

* BVS were calculated using the following parameters: U [32], P, Ca, Mn [33].

## Data Availability

Not applicable.

## Supplementary Materials

The following supporting information can be downloaded at: https://www.mdpi.com/article/10.3390/ma16134533/s1, CSD 2256603-2256605 for **Bqr_1**, **Bqr_2**, and **Phu**, respectively: contain the supplementary crystallographic data for this paper.
